# The Feasibility of Using Computrition Software for Nutrition Research—A Pilot Study

**DOI:** 10.3390/nu13020329

**Published:** 2021-01-23

**Authors:** Courtney L. Millar, Alegria Cohen, Stephen P. Juraschek, Abby Foley, Misha Shtivelman, Kenneth J. Mukamal, Shivani Sahni

**Affiliations:** 1 Hinda and Arthur Marcus Institute for Aging Research, Roslindale, Boston, MA 02131, USA; courtneymillar@hsl.harvard.edu (C.L.M.); afole89@gmail.com (A.F.); 2 Hebrew Senior Life, Roslindale, Boston, MA 02131, USA; alegriacohen@hsl.harvard.edu (A.C.); mishashtivelman@hsl.harvard.edu (M.S.); 3 Department of Medicine, Beth Israel Deaconess Medical Center, Harvard Medical School, Boston, MA 02115, USA; sjurasch@bidmc.harvard.edu (S.P.J.); kmukamal@bidmc.harvard.edu (K.J.M.)

**Keywords:** sodium, diet, pilot, nutrition, aging, older adults, falls

## Abstract

We evaluated the feasibility of using Computrition to design and implement a low vs. typical sodium meal plan intervention for older adults. Dietitians used Computrition to design a 7-day meal plan with three caloric levels (≤1750, 2000, ≥2250 kcals/day) and two sodium densities (low = 0.9 mg/kcal; *n* = 11 or typical = 2 mg/kcal; *n* = 9). Feasibility was determined by *post-hoc* definitions of effectiveness, sodium compliance, palatability of diet, sustainability, and safety. Given the low number of participants in one of the three calorie groups, the higher calorie groups were combined. Thus, comparisons are between low vs. typical meal plans at two calorie levels (≤1750 or ≥2000 kcals/day). Overall, regardless of the calorie group, the meal plans created with Computrition were effective in reaching the targeted sodium density and were safe for participants. Furthermore, individuals appeared to be equally compliant and reported similar palatability across meal plans. However, one of the three criteria for the sustainability definition was not met. In conclusion, we successfully used Computrition to design low and typical sodium meal plans that were effective, compliable, and safe. Future studies of older adults in similar settings should focus on improving the palatability of the meal plans and scaling this protocol to larger studies in older adults.

## 1. Introduction

Most nutritional feeding studies examining the effects of diet on health outcomes have enrolled individuals less than 65 years old, and few studies have incorporated older populations. Independent living facilities, which house older adults, offer a unique opportunity to conduct interdisciplinary nutrition feeding studies. At such facilities, older adults often rely on others for meal preparation, which provides nutritional and social support [1]. Such meal programs present a convenient means to conduct a well-controlled, blinded feeding study in an older population.

Despite their seeming attractiveness, designing and implementing dietary interventions in such settings is often faced with seemingly insurmountable difficulties. These barriers include labor-intensive processes [2], the poor participant compliance or low palatability of the diet leading to high rates of attrition [3], the necessity of a collaborative research team including administrative staff, kitchen staff, dietitians, clinicians, and researchers [4], the high costs of dietary trials, ineffective blinding [5], and limited funding mechanisms. Given the growing interest in lifestyle changes for disease prevention [4], a particular need exists for established methodologies and well-designed protocols to serve as a platform for effectively implementing dietary interventions; one that targets the aforementioned barriers to improve the quality and robustness of nutritional feeding studies, particularly in older adults. 

Computrition is a proprietary food service software package used extensively by dietitians in hospitals and long-term care facilities [6]. This software sources foods from various vendors and catalogs them with their corresponding nutritional information. Dietitians use this software to create standardized recipes, design menus, and then scale these menus to larger quantities, making it an excellent choice for designing nutrition interventions with the goal of scaling them to larger studies. However, to our knowledge, this software has not been utilized in the design of dietary interventions for clinical research. 

The objective of this study was to determine the feasibility of administering two meal plans (low sodium vs. typical sodium density) at two different calorie levels (≤1750 or ≥2000 kcals/day) designed with Computrition in a double-blind, randomized pilot study in older adult residents of a congregate housing facility. We hypothesized that it would be feasible to implement a dietary sodium intervention with meal plans designed by Computrition, and that feasibility measures would not differ by calorie group. 

## 2. Materials and Methods 

### 2.1. The Satter House Trials of Reduced Sodium Study

The Satter House Trials of Reduced Sodium Meals (SOTRUE) study was a double-blind, randomized, controlled pilot trial of 19 females and 1 male aged ≥65 years residing in a congregate living facility for older adults. This trial was registered on clinicaltrails.gov (NCT04074941) and the study protocol was approved by the Institutional Review Board at the Hinda and Arthur Marcus Institute for Aging Research, Hebrew SeniorLife. SOTRUE participants were randomized in a 1:1 ratio to either a low sodium (<0.95 mg/kcal/day; *n* = 11) or typical sodium (>2 mg/kcal/day *n* = 9) meal plan. The primary outcome of the SOTRUE study was to determine the effect of two diets on seated blood pressure over 14 days. Each day participants received breakfast, lunch, dinner, and two snacks to consume. Our objective was to determine the feasibility of implementing the dietary sodium intervention designed by Computrition that was used in the SOTRUE study.

### 2.2. Menu Design

A base 7-day meal plan was initially developed by the executive chef. First, culturally sensitive entrees and snacks were designated for each day to provide a variety of foods for the participants. Once the base meal plan was designed, the corresponding recipes developed by the executive chef were entered into the Computrition software. The study dietitian then modified the recipes to achieve the two target sodium densities by adding or removing sodium from the recipes. Then, the recipes were adjusted to meet three caloric levels (~1750, 2000, and 2250 kcal per day), based on historic calorie needs in our population, while keeping macronutrients constant. Caloric appropriateness was achieved by adding or removing energy-dense foods, such as cookies or crackers, that were also low in sodium to maintain target sodium densities. Given that potassium intake affects blood pressure (the primary outcome) [7], potassium levels were kept constant at roughly the adequate intake for adults over 51 years old (e.g., ~3500 mg/day). Once the base menu was finalized, it was repeated for the second week of the study.

### 2.3. Randomization and Blinding

Participants were randomized to the two diets to achieve a balanced number for each study arm. To maintain blinding throughout the study, the research team and study participants were unaware of the diet assignments. Only the kitchen staff, who prepared the meals, knew the sodium diet assignments.

### 2.4. Menu Implementation

Once the meal plan was finalized, participants were allocated to a caloric level based on their estimated caloric requirements. For each participant, individual caloric needs per day were estimated using the Mifflin St. Jeor calculation [8], which is based on an individual’s age, body mass index (BMI, kg/m^2^), and self-reported physical activity. Physical activity was evaluated using the Godin–Shepard Leisure-Time Questionnaire [9], which was administered to participants before randomization. On the day of randomization, as well as the day before the study intervention, weight (pounds) was measured twice using a digital scale and then averaged. Height (cm) was measured twice on the day of randomization using a stadiometer. BMI was calculated by dividing the weight (kg) by height (m^2^). 

Once the participants were assigned a caloric level and randomized to one of the intervention arms, Computrition software was used to generate meal tickets for each participant. These tickets denoted the exact recipe and portion for each component of the meals. These meal tickets were used to prepare the meals for each participant while maintaining blinding by limiting the access of this information to only the kitchen. Each day, the participants received 2 deliveries of the prepared meals to their apartment. One delivery in the morning contained their breakfast, lunch, and one snack and the afternoon delivery contained their dinner and remaining snack. Despite home-delivery, participants had the option to consume their meals in the dining room, to maintain the normality of their usual lifestyle.

### 2.5. Baseline Characteristics

Before the intervention, demographic information (age, sex, and ethnicity) and medical history were obtained via self-reported questionnaires. This included medication use, history of medical conditions, physical activity, and allergies.

### 2.6. Feasibility

To determine the feasibility of using Computrition software in a clinical setting, the effectiveness, compliance, safety, palatability, and sustainability were evaluated. To be considered entirely feasible, we required that 5 criteria (effectiveness, compliance, safety, palatability, and sustainability) be met, whereas meeting 3 of the 5 criteria were considered moderately feasible, with improvements needed. Anything less than 3 criteria was considered infeasible. Since some of our measures of feasibility (e.g., palatability, food waste, and compliance) could have differed at various caloric levels, all comparisons were assessed in sub-groups by caloric needs (≤1750 kcal/day, *n* = 12 and ≥2000 kcal/day, *n* = 8).

Effectiveness was defined as a percent difference of <5% between targeted vs. prepared sodium densities. The percent difference between targeted and prepared sodium densities was calculated by the equation: [(prepared sodium density—targeted sodium density)/targeted sodium density] × 100%. 

Compliance was defined as meeting one of the following requirements: 1. no difference in the percentage of provided sodium consumed when comparing the low vs. typical sodium meal plans, and 2. a significant difference in pre- to post-change in urinary sodium when comparing the low vs. typical sodium meal plans. Each day, participants were asked to document the percentage of each meal/snack consumed. To determine how compliant the individuals were for their targeted sodium intake, the self-reported meal consumption percentage was multiplied by the corresponding meals’ sodium percentage for that given day. To derive a daily percentage of targeted sodium intake, all the meals and snacks were totaled together. Finally, to calculate an individual’s overall percentage of provided sodium consumed, an individual’s sodium intake percentage for all days on study meals was averaged. Urinary sodium (mmol/L; a surrogate marker for sodium intake), potassium (mmol/L) and creatinine (mg/dL) were measured by Quest Diagnostics. Participants were asked to provide a urine sample pre- and post-intervention. Compliance was measured by comparing the change in urinary sodium over 14 days between the low sodium and typical sodium groups at each calorie level.

At post-intervention exit interviews, participants were asked about the occurrence of any adverse events, as well as the overall palatability of the diet. Safety was evaluated by the number of adverse events reported by participants. If there were any adverse events reported during the study, then the respective meal plan was considered not safe.

Palatability was determined by participants’ self-reported willingness to continue the diet long-term. At the exit interview, participants were asked to rate the statement, “I would eat this diet long-term,” on a scale from 1 to 5 (with 1 defined as “none of the time/never” and 5 defined as “all of the time/always”). Those that responded with 1 or 2 to that statement were considered “unlikely to continue the diet long-term,” whereas responses of 3, 4, and 5 were considered “likely to continue the diet long-term”. The percentage of people likely to continue the diet long-term by calorie group and meal plan was calculated. A palatable meal plan was defined as >75% of individuals reporting that they were likely to continue their specific diet long-term.

The long-term sustainability of this intervention was determined by 3 different factors: reports of food waste, blinding efficacy, and the number of delivery errors. During the exit interview, participants were asked to rate the question, “How often did you waste or store food because you had too much?” on a scale from 1 to 5 (with 1 defined as “none of the time/never”, and 5 defined as “all of the time/always”). Those that responded with 1, 2, or 3 to that question were considered “unlikely to waste food,” whereas responses of 4 or 5 were considered “likely to waste food.” The percentage of individuals reporting they were likely to waste food was calculated. Blinding efficacy was evaluated by recording the participant’s guess for which sodium meal plan they were allocated to following the intervention. The percentage of individuals guessing assignment to the low sodium diet was calculated by the meal plan and calorie group. As a final measure of sustainability, the kitchen staff documented any reports of meal delivery errors. To be considered sustainable long-term, the three following criteria had to be met: 1. <25% of individuals were likely to waste food in either meal plan; 2. the percentage of people guessing assignment to the low sodium group was not significantly different between meal plans; and 3. delivery errors had to be less than 1% of total deliveries.

### 2.7. Statistical Analysis

Given the low number of participants in one of the 3 calorie groups (≥1750, *n* = 12; ≥2000, *n* = 3; ≥2250 kcal, *n* = 5), the higher calorie groups were combined. Thus, the results are presented for two calorie groups alone (≤1750 or ≥2000 kcal). The data are presented as intent to treat, except for the question on food waste. Because two individuals stopped consuming study meals, they could not answer the question of how much food was wasted; this question is presented per protocol.

Descriptive variables were compared using a student *t*-test or Fisher’s exact test between the two diets within each caloric level. For both meal plans, the targeted and prepared values for specific nutrients (total energy, kcal; percent carbohydrates, %; total sodium, mg/day; sodium density, mg/kcal/day; and total potassium, mg/day) were calculated. The percent difference between the targeted and prepared values for specific nutrients was calculated to assess the effectiveness of the intervention. Mean values for compliance measures were compared using a student *t*-test between the two diets by calorie levels. Differences between palatability and sustainability were determined by a two-sided Fisher’s exact test. Given the small sample size and pilot nature of the trial, we set a *p*-value threshold of 0.25 as suggestive of differences, as recommended [10]. All statistical analyses were performed using SAS software version 9.4. 

## 3. Results

### 3.1. Menus

The 7-day meal plan as designed by the Computrition software is shown in Table 1. The percentage of sodium in each meal-by-meal plan and caloric level is shown in Supplementary Table S1.

### 3.2. Baseline Characteristics

The mean ages of SOTRUE participants were 79.5 years (SD 9.4) on the typical sodium meal plan and 77.0 years (SD 6.2) for those on the low sodium meal plan (Supplementary Table S2). Baseline characteristics by meal plan and calorie group are shown in Table 2.

### 3.3. Effectiveness

The percent difference between the targeted and prepared nutrient values, by calorie group, are presented in Table 3. The percent differences between prepared and targeted sodium densities were all less than 5% (range: 2–4%), regardless of meal plan or calorie group.

### 3.4. Compliance

Overall, compliance rates as assessed by the self-reported mean percentage of sodium consumed were moderate, ranging from 54 to 73% (Table 4). We observed no differences in the compliance rates between the low versus typical sodium diets in either stratum of energy intake, as assessed by the percentage of sodium consumed, urinary potassium, or creatinine. Urinary sodium decreased by 18.3–22.0 mmol/L in the two lower-sodium diets, with no comparable change in the typical sodium diets.

### 3.5. Safety

There were no reported adverse events for the entire duration of the intervention. However, two individuals stopped consuming study meals; one after 4 days of being on the diet, and the other after 7 days. Both individuals raised concerns about the effect of the study meals on their blood sugar and were diabetic/pre-diabetic. While both individuals were randomized to the typical sodium meal plan, one was allocated to the lower calorie meal plan (≤1750 kcal/day), while the other was allocated to the higher calorie meal plan (≥2000 kcal/day).

### 3.6. Palatability

**Figure 1:**
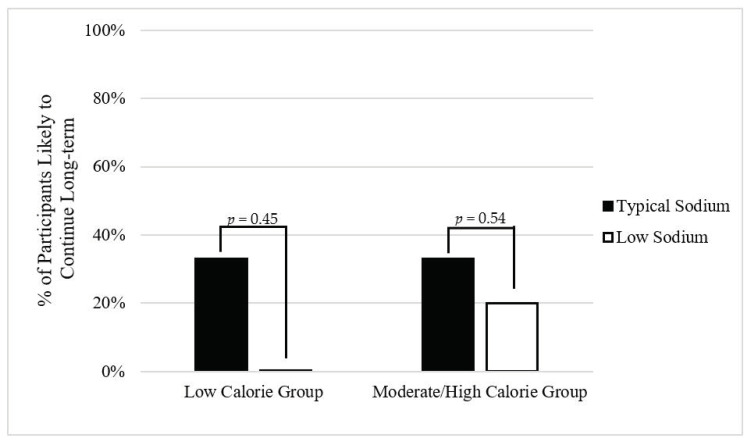
Self-reported willingness to continue meal plans long-term by diet and calorie group. Twelve participants were in the low calorie group (typical sodium, *n* = 6; low sodium, *n* = 6), and eight participants were in the moderate/high calorie group (typical sodium, *n* = 3; low sodium, *n* = 5).

Overall, the majority of participants reported they would not be willing to follow the diet long-term (Figure 1). However, there were no significant differences in palatability between the low and typical sodium diets, regardless of calorie level.

### 3.7. Sustainability

Out of 526 deliveries, only 2 (0.4%) delivery errors occurred, which were immediately corrected. Table 5 shows the other two metrics of sustainability. 

#### 3.7.1. Low Calorie Group

No individuals on the typical sodium meal plan reported that they were likely to waste food. However, 16.7% of those on the low sodium meal plan were likely to waste food. For blinding efficacy, 67% of individuals in both the typical and the low sodium meal plans guessed they were assigned the low sodium meal plan.

#### 3.7.2. Moderate/High calorie Group

No individuals on the typical sodium meal plan reported that they were likely to waste food. However, 20% of those on the low sodium meal plan reported that they were likely to waste food. While 60% of individuals on the low sodium meal plan guessed they were assigned to the low sodium diet, no individuals on the typical meal plan did so (*p* = 0.09). 

## 4. Discussion

Overall, the use of Computrition software to design a nutrition intervention was moderately feasible based on our pre-specified criteria for effectiveness, compliance, and safety. It enabled us to design low sodium meal plans at three different caloric levels, all within 5% of the target while keeping potassium constant. The compliance with the meal plans appeared to be moderate overall, with no differences between the percentages of provided sodium consumed between the two sodium meal plans. These meal plans were also deemed safe in this cohort of individuals. Despite overall low palatability, low and typical sodium meal plans were similar, suggesting that sodium content was not the driver of palatability. Further, these meal plans were moderately sustainable long-term given that two of three criteria (i.e., food waste and delivery errors) for sustainability were met. 

The use of Computrition software in research studies is limited. Most often, it is used to calculate the nutrient intakes of participants [11,12,13]. It has also been used in studies to estimate food costs of recipes [14] or the overall quality of online meal ordering systems [15]. Nonetheless, its greatest use is in non-research settings. Hospitals and nursing facilities often use the software to design recipes/daily menus to meet appropriate nutrient intakes of patients, and also to scale them to desired quantities, which are often important goals when designing well-controlled dietary interventions. Moreover, Computrition can map food brands, making it possible to standardize menus, which would be a valuable feature for multi-site studies, but its practicality in the research setting is unknown. 

In our study, Computrition software was used to do the following: 1. design daily recipes targeting specific sodium densities (0.9 mg/kcal and 2.0 mg/kcal); 2. scale these recipes to the desired number of participants; and 3. confirm the sodium densities of our prepared meals. The prepared sodium densities were within 5% of the targeted values, suggesting that using Computrition was effective in designing two meal plans with varying concentrations of sodium. However, it is important to note that the prepared potassium was within 15–25% of the targeted potassium levels (Table 3). When evaluating the effect of dietary sodium, it is critical to maintaining an appropriate sodium/potassium ratio since it can also affect blood pressure [16]. Our dietitians found it challenging to maintain a variety of foods and also reach the targeted potassium levels, since many foods with substantial potassium content (e.g., bananas, potatoes) are also higher in calories. Thus, we encountered a trade-off between reaching the targeted calorie percentage or the target potassium, highlighting the difficulty in designing sodium diets with the appropriate levels of other nutrients and calories simultaneously. Given that the actual potassium level was consistently lower than the targeted level in the diets, future studies could consider including supplemental potassium to reach both the desired calorie and potassium levels. 

Overall, participants were relatively compliant across meal plans and calorie groups, with similar numbers reporting between 50 and 70% for sodium intake compliance. The original study on the dietary approaches to stop hypertension (DASH) diet also reported variable compliance [17]. Additionally, our population was accustomed to eating a self-prepared breakfast and lunch, with dinner prepared by the on-site kitchen staff, which may have influenced compliance. Some participants anecdotally mentioned in exit interviews that they were not used to eating “that much” food. Since self-reported measures are subject to misreporting, we also evaluated spot urinary sodium as a biological measure of sodium compliance. Those on the low sodium meal plan did have larger reductions in urinary sodium compared to those on the typical sodium meal plan, which corroborates that these individuals were relatively compliant for sodium intake. Interestingly, studies have reported that a phone-delivered behavioral intervention can be effective in improving adherence to a low sodium diet [18], which may be a useful strategy to improve compliance in future dietary sodium interventions.

Most low sodium feeding studies do not specifically address palatability, despite its importance as a determinant of long-term adherence [19]. During aging, the ability to taste diminishes, often resulting in an increased preference for salty foods [20]. Thus, one would anticipate that a low sodium meal plan would be less palatable compared to a typical sodium meal plan. Instead, our study found that the long-term palatability of both meal plans was relatively low. Since both meal plans were not preferred by the participants, it appears that sodium content was not the driving force of low palatability. However, given that this is a pilot study, studies with larger sample sizes are needed to confirm these findings. In addition to the palatability of the food, the quality of the food management services is a major determinant of customer satisfaction in assisted living facilities [21]. It is possible that participants’ baseline satisfaction with their usual meal plans was relatively low, but this was not measured. To overcome this challenge, researchers should consider a pre-study trial period to test the palatability of the designed meal plans. Another strategy to increase palatability would be to provide more than one option per day. Providing older adults with options is an important aspect of preserved autonomy in the aging population [22], and may improve food intake [23]. Thus, additional option offerings should be considered in future interventions. 

In addition to compliance and palatability, the safety of a diet is of the utmost importance for older adults. Reductions in sodium can increase the incidence of hypotension, which could increase the likelihood of falls. The results of this study suggest that the designed meal plans with lower sodium were safe for this population of older adults, at least at the sodium levels tested. Similarly, Hummel et al. [24] reported in their study of a low sodium (~1500 mg/day) home-delivery meal program in older heart-failure patients that it was safe since reports of adverse events were rare. 

There were several strengths to this study. Our study team was equipped with a highly-skilled, interdisciplinary team that consisted of full kitchen staff, executive chef, registered dietitians, clinicians, and research staff. Additionally, this was a randomized, double-blind study that assessed the feasibility of a low sodium meal plan in older adults, which is often an under-researched population. Along with our strengths, there were limitations, such as a small number of participants and short follow-up time. Due to the low number, it is unclear if participants were appropriately blinded in the higher calorie group since all participants on the typical meal plan accurately guessed their dietary assignment. Thus, larger trials are warranted. Furthermore, our results are not generalizable to other populations, including younger adults and older adults living outside congregate housing facilities. 

## 5. Conclusions

Overall, this study suggests that the use of Computrition was moderately feasible given its effectiveness, compliance, and safety in a dietary sodium intervention for older adults residing in a congregate housing facility. Although palatability was low on both meal plans, it was not affected by the level of sodium. Furthermore, both the low and typical sodium diets appeared to be potentially sustainable given that two of the three criteria of the sustainability definition were met. However, larger studies are needed to confirm the blinding efficacy of the diets. Strategies to improve the study infrastructure include the inclusion of a pre-study trial of the proposed meal plans, the use of food options to preserve autonomy or providing only one meal per day, which may improve the palatability and sustainability of the meal plans. Future studies should aim to confirm the feasibility of utilizing Computrition in dietary interventions on a larger scale and in more diverse populations. 

## Figures and Tables

**Table 1:** The 7-day meal plan for the low and typical sodium groups in the SOTRUE study.

	Day 1	Day 2	Day 3	Day 4	Day 5	Day 6	Day 7
**Breakfast**	Eggs Bread Orange Juice	English Muffin Peanut Butter and Jelly Egg Fruit cup Juice	Cereal Yogurt Banana Juice	Muffin Cottage Cheese Banana Juice	Bagel Cream CheeseYogurt Juice	Muffin Cottage Cheese Fruit Juice	Omelet Bagel Cream Cheese Yogurt Banana Juice
**Lunch**	Turkey Burger Potato Salad Pear	Pasta Primavera Breadstick Salad w/dressing	Tuna Salad Wrap Pasta Salad Cookies Mandarin Orange	Whitefish Salad Plate w/dressing Pita Bread Israeli Salad	Chicken Salad Sandwich Pasta Salad Peaches Applesauce	Quiche Homefries Mandarin Oranges	Grilled Chicken Salad Plate w/dressing Potato Chips
**Snack 1**	Cookies Water	Pudding Water	Crackers Water	Pudding Water	Crackers w/peanut butter Water	Pudding Water	Cookie Water
**Snack 2**	Cookies Water	Peaches Water	Cookie Water	Cookie Water	Cookie Water	Jello Water	Fruit Water
**Dinner**	Salad w/dressing Baked Fish Rice Spinach	Salad w/dressing Brisket Potatoes Carrots	Salad w/dressing Chicken Kabob Rice	Salad w/dressing Roast Chicken Mashed Sweet Potato Corn Apple	Salad w/dressing Spaghetti and Meatballs Roll Fruit	Salad w/dressing Salmon Burger Asparagus	Salad w/dressing Meatloaf w/gravy Potatoes Green Beans

**Table 2:** Baseline characteristics of SOTRUE participants by meal plan and calorie group.

	Low Calorie Group (*n* = 12)			Moderate/High Calorie Group (*n* = 8)		
	Typical Sodium (*n* = 6)	Low Sodium (*n* = 6)	*p*-Value	Typical Sodium (*n* = 3)	Low Sodium (*n* = 5)	*p*-Value
Age (year, range 64–91)	82.2 ± 9.4	80.6 ± 5.3	0.74	74.3 ± 8.5	72.6 ± 4.2	0.71
Female, *n* (% ^a^)	6 (100)	6 (100)	1.00	3 (100)	4 (80)	0.41
European ancestry, *n* (%)	6 (100)	5 (83)	0.29	3 (100)	5 (100)	1.00
Height (cm)	152.3 ± 2.5	159.2 ± 6.0	0.03 *	158.6 ± 7.0	156.1 ± 6.6	0.64
Weight (kg)	65.6 ± 13.4	70.0 ± 7.8	0.50	99.0 ± 13.4	92.3 ± 16.5	0.63
BMI (kg/m^2^)	28.3 ± 5.9	27.6 ± 2.6	0.80	40.0 ± 6.8	38.4 ± 7.3	0.82
Caloric Intake (kcal/day)	1599 ± 152	1669 ± 111	0.34	2158 ± 130	2065 ± 163	0.85
Physical Activity Score	27.5 ± 12.5	16.3 ± 14.0	0.18 *	13.3 ± 12.6	15.4 ± 18.0	0.87
Current Smoking, *n* (%)	0 (0)	2 (33)	0.12 *	0 (0)	0 (0)	1.00
Hypertensive Medication, *n* (%)	2 (33)	3 (50)	0.56	3 (100)	4 (80)	0.41
CVD Conditions, *n* (%)	5 (84)	4 (67)	0.51	1 (33)	3 (60)	0.47
GI Conditions, *n* (%)	0 (0)	1 (17)	0.29	1 (33)	2 (40)	0.85
Diabetes, *n* (%)	2 (33)	0 (0)	0.12 *	1 (33)	3 (60)	0.47
Cancer, *n* (%)	2 (33)	0 (0)	0.12 *	0 (0)	1 (20)	0.41

Physical activity score: Range (0–42). * Representative of *p* < 0.25 was pre-specified as statistically significant. ^a^ Representative of column specific percentages.

**Table 3:** The targeted and the prepared nutrient values by meal plan and calorie group.

	Typical Sodium			Low Sodium		
	Targeted	Prepared	% Difference	Targeted	Prepared	% Difference
	**Low Calorie Group**					
Energy, kcal/day	1750	1861	6.3	1750	1842	5.3
Carbohydrates, % ^1^	50	52	4.0	50	49	−2.0
Sodium, mg/day	3500	3589	2.5	1650	1643	−0.4
Sodium Density, mg/kcal	2.00	1.93	−3.5	0.95	0.98	3.2
Potassium, mg/day	3500	2953	−15.6	3500	2966	−15.3
	**Moderate/High calorie Group**					
Energy, kcal/day	2125	2177	2.4	2125	2072	−2.5
Carbohydrates, % ^1^	50	52	4.0	50	50	−1.0
Sodium, mg/day	4250	4233	−0.4	2000	2008	0.4
Sodium Density, mg/kcal	2	2	−2.8	1	1	2.1
Potassium, mg/day	4250	3271	−23.0	4250	3140	−26.1

^1^ Carbohydrate intake as a percentage of energy per day.

**Table 4:** Self-reported and urinary compliance by meal plan and calorie group.

	Low Calorie Group			Moderate/High Calorie Group		
Compliance Measure	Typical Sodium (*n* = 6)	Low Sodium (*n* = 6)	*p*-Value	Typical Sodium (*n* = 3)	Low Sodium (*n* = 5)	*p*-Value
Percentage of Provided Sodium Consumed (%)	61.1 ± 24.3	73.6 ± 22.4	0.37	54.0 ± 40.9	68.2 ± 22.5	0.54
Δ Sodium (mmol/L)	−2.3 ± 22.2	−18.3 ± 12.6	0.16*	0.0 ± 12.5	−22.0 ± 28.2	0.26
Δ Potassium (mmol/L)	−1.0 ± 25.9	4.0 ± 33.4	0.78	5.0 ± 18.4	7.6 ± 24.6	0.88
Δ Creatinine (mg/dL)	−5.83 ± 73.2	6.7 ± 19.0	0.69	−27.7 ± 40.1	−1.4 ± 38.3	0.39

Δ = change between baseline and follow-up. * = *p* < 0.25 was pre-specified as statistically significant.

**Table 5:** Sustainability of meal plans by calorie group.

	Low Calorie Group			Moderate/High Calorie Group		
	Typical Sodium (*n* = 6)	Low Sodium (*n* = 6)	*p*-Value	Typical Sodium (*n* = 3)	Low Sodium (*n* = 5)	*p*-Value
Likely to Waste Food (%)	0%	16.7%	0.55	0%	20%	0.71
Guessed Low Sodium Diet Assignment (%)	67%	67%	1.00	0%	60%	0.09 *

* = *p* < 0.25 was pre-specified as statistically significant.

## Supplementary Materials

The following are available online at https://www.mdpi.com/2072-6643/13/2/329/s1, Table S1: Targeted sodium percentage for each meal by diet and calorie level, Table S2: Baseline characteristics of all participants by meal plan.
