# Supplementary material for: The Feasibility of Using Computrition Software for Nutrition Research—A Pilot Study
Source: Nutrients. 2021 Jan 23;13(2):329. doi: 10.3390/nu13020329 (PMC7911746; doi:10.3390/nu13020329)
Supplement: Supplementary file 1 [file nutrients-13-00329-s001.pdf]

Supplemental Table S1. Targeted sodium percentage for each meal by diet and calorie level.

Percentage Sodium for Low Sodium, 1750kcal								Percentage Sodium for Typical Sodium, 1750kcal							
	Day 1	Day 2	Day 3	Day 4	Day 5	Day 6	Day 7		Day 1	Day 2	Day 3	Day 4	Day 5	Day 6	Day 7
Breakfast	18%	22%	17%	8%	26%	10%	32%	Breakfast	10%	11%	6%	14%	14%	26%	20%
Lunch	34%	43%	57%	24%	44%	43%	23%	Lunch	15%	39%	34%	34%	27%	30%	36%
Snack 1	5%	0%	8%	7%	7%	1%	12%	Snack 1	1%	0%	3%	4%	4%	0%	3%
Dinner	42%	35%	9%	60%	23%	39%	33%	Dinner	74%	49%	54%	44%	56%	41%	42%
Snack 2	0%	0%	8%	0%	0%	7%	2%	Snack 2	0%	0%	3%	4%	0%	3%	0%
**Percentage Sodium for Low Sodium, 2000kcal**								**Percentage Sodium for Typical Sodium, 2000kcal**							
	Day 1	Day 2	Day 3	Day 4	Day 5	Day 6	Day 7		Day 1	Day 2	Day 3	Day 4	Day 5	Day 6	Day 7
Breakfast	14%	20%	13%	8%	23%	29%	29%	Breakfast	9%	9%	6%	14%	12%	21%	15%
Lunch	35%	47%	68%	29%	39%	34%	21%	Lunch	19%	41%	39%	39%	23%	38%	31%
Snack 1	4%	0%	6%	7%	11%	2%	6%	Snack 1	2%	0%	3%	3%	6%	0%	3%
Dinner	42%	32%	7%	48%	26%	31%	42%	Dinner	67%	50%	49%	40%	60%	38%	51%
Snack 2	5%	0%	6%	7%	0%	5%	4%	Snack 2	3%	0%	3%	4%	0%	2%	1%
**Percentage Sodium for Low Sodium, 2250kcal**								**Percentage Sodium for Typical Sodium, 2250kcal**							
	Day 1	Day 2	Day 3	Day 4	Day 5	Day 6	Day 7		Day 1	Day 2	Day 3	Day 4	Day 5	Day 6	Day 7
Breakfast	18%	17%	11%	8%	20%	25%	30%	Breakfast	9%	8%	5%	20%	11%	20%	16%
Lunch	40%	56%	72%	36%	35%	30%	35%	Lunch	20%	46%	42%	37%	28%	36%	37%
Snack 1	5%	0%	5%	0%	10%	2%	5%	Snack 1	2%	0%	3%	3%	5%	0%	1%
Dinner	33%	27%	7%	48%	30%	40%	28%	Dinner	67%	45%	48%	36%	53%	42%	44%
Snack 2	5%	0%	5%	7%	5%	5%	2%	Snack 2	3%	0%	2%	3%	3%	2%	2%

Supplemental Table S2. Baseline characteristics of all participants by meal plan.

	**All Participants (n=20)**		
	Typical Sodium (n=9)	Low Sodium (n=11)	P-value
Age (y, range 64-91)	79.5 ± 9.4	77.0 ± 6.2	0.48
Female, n (%)	9 (100)	10 (91)	0.35
Caucasian, n (%)	9 (100)	10 (91)	0.35
Height (cm)	154.4 ± 5.1	157.8 ± 6.2	0.21*
Weight (kg)	76.7 ± 20.9	80.6 ±16.9	0.65
BMI (kg/m^2)	32.1 ± 8.1	32.5 ± 7.5	0.90
Caloric Intake (kcal/d)	1785 ± 322	1849 ± 235	0.61
Physical Activity Score	22.8 ± 13.7	15.9 ± 15.1	0.31
Current Smoking, n (%)	0 (0)	2 (18)	0.18*
Hypertensive Medication, n (%)	5 (56)	7 (64)	0.71
CVD Conditions, n (%)	7 (64)	6 (67)	0.89
GI Conditions, n (%)	1 (11)	3 (27)	0.37
Diabetes, n (%)	3 (33)	3 (27)	0.77
Cancer, n (%)	2 (22)	1 (9)	0.41

*= $p<0.25$ was pre-specified as statistically significant
